# The impact of generative artificial intelligence (AI) on the development of personalized pharmaceuticals and the future of precision medicine

**DOI:** 10.17179/excli2024-7915

**Published:** 2024-11-26

**Authors:** Varghese Edwin Hillary, Rajiv Gandhi Gopalsamy, Lucas Alves da Mota Santana, Pamela Chaves de Jesus, Jessiane Bispo de Souza, Deise Maria Rego Rodrigues Silva, Pedro Henrique Macedo Moura, Ronaldy Santana Santos, Marina dos Santos Barreto, Lysandro Pinto Borges, Eloia Emanuelly Dias Silva

**Affiliations:** 1 Division of Biotechnology, Department of Biosciences, Rajagiri College of Social Sciences (Autonomous), Kochi, Kerala, India; 2 Division of Phytochemistry and Drug Design, Department of Biosciences, Rajagiri College of Social Sciences (Autonomous), Kochi, Kerala, India; 3 Department of Dentistry, Federal University of Sergipe (UFS), Aracaju, Sergipe, Brazil; 4 Department of Pharmacy, Federal University of Sergipe (UFS), Aracaju, Sergipe, Brazil; 5 Department of Biology, Federal University of Sergipe (UFS), Aracaju, Sergipe, Brazil

## ⁯⁯

Personalized medicine is at the forefront of revolutionary advancements in disease prevention, diagnosis, and management within the rapidly evolving healthcare landscape (Abrahams and Downing, 2023[2]). A comprehensive understanding of individual genetic, environmental, and lifestyle variability enables personalized therapeutic approaches. The advancement of high-throughput, data-intensive biomedical research tools and tests - such as genomics, metabolomics, transcriptomics, proteomics, molecular interactions, medical imaging, and wireless health monitoring devices - requires researchers to develop novel strategies to analyze, integrate, and interpret the vast amounts of medical data being generated (Zhan et al., 2023[15]). In the past decade, breakthroughs in computational biology, algorithms, and big data have driven the widespread use of artificial intelligence (AI) across all major fields (Gou et al., 2024[6]). Consequently, AI is expected to play a pivotal role in advancing precision medicine. This letter highlights the potential of generative AI in the development of personalized pharmaceuticals and the future of precision medicine.

AI is a powerful tool that leverages personal data to provide faster solutions to complex problems. The application of AI algorithms in the pursuit of precision medicine shows great potential. These algorithms have expanded our understanding of disease mechanisms by uncovering dysregulated molecular pathways, predicting novel therapeutic targets, and evaluating the in silico efficacy of drugs (Johnson et al., 2021[7]). Moreover, the AI-driven decision-making process supports the exploration of three key elements: big data, multimodal assessments, and decision support systems.

Owing to these distinctive characteristics, AI has become a valuable resource in healthcare, presenting significant opportunities for medical progress and study (Ali and Mohammed, 2024[3]). For instance, ***1)*** ***AI-powered diagnosis of disease:*** The use of AI in illness diagnostics has markedly improved accuracy, efficiency, and efficacy. AI examines extensive medical data, including patient records and genetic profiles, identifying complex relationships that may elude human detection (Luz and Ray, 2024[8]). In addition, AI in dermatology attained a remarkable accuracy rate of 95%, outperforming dermatologists, which obtained 86.6%, using a dataset of 130,000 images. This remarkable achievement enhances medical practitioners' capabilities, significantly improving the precision and effectiveness of disease diagnosis and prognosis (Luz and Ray, 2024[8]). ***2)*** ***Identifying potential drug targets:*** AI in identifying potential drug targets plays a pivotal role in pinpointing drug targets crucial in the regression of disease (Sharma et al., 2024[10]). AI algorithms evaluated vast biological and genetic datasets, revealing novel targets for therapies. Notably, in cancer research, AI successfully examined data from thousands of patients in association with the Cancer Genome Atlas (Sharma et al., 2024[10]). ***3)*** ***Identifying causal genes:*** Despite the challenge of accurately detecting common missense mutations, AI techniques play a crucial role in identifying causal genes and loci (Wu and Xie, 2024[14]). In addition, bioinformatics tools struggle to distinguish dangerous mutations from "Variations of Uncertain Significance" (VUS). To address this, AI shows potential in improving diagnosis of disease especially neurodevelopmental diseases (NDDs) (Thapliyal and Thapliyal, 2024[11]).

Notably, the Human Splicing Code and DeepSEA algorithms have performed exceptionally well in identifying missense variants. This represents a significant advancement, as they demonstrate an 85 % improvement in accuracy, highlighting AI's potential to revolutionize the identification of genetic disorders and the development of personalized therapies (Uddin et al., 2019[12]). Moreover, AI can help address complex genetic variants, marking a new era in genomics research and precision medicine. ***4) Phenotypic and genetic heterogeneity:*** Disorders such as NDDs are predominantly inherited, yet environmental factors modulate disease severity by altering hereditary neural pathways (Zucca et al., 2024[16]). Mutations like postzygotic mosaicism, associated with NDDs, also contribute to intellectual disability, epilepsy, and autism spectrum disorders. Future therapeutic approaches will benefit from the digitalization of these large healthcare datasets. Thus, applying AI systems to link genetic information with other characteristics can significantly enhance disease research and propel pivotal advances in NDD studies. Additionally, the complex landscape of NDDs is further complicated by environmental effects on hereditary brain patterns, emphasizing the need for extensive datasets and advanced technologies (Vuong, 2024[13]). Consequently, employing AI methodologies with digital medical data has the potential to transform both NDD research and management in the future.

Despite the immense potential advantages of generative AI for the pharmaceutical industry, several challenges still require resolution (Gangwal et al., 2024[5]). For example, the ethical application of AI algorithms in pharmaceutical development and patient care must be addressed. Although AI has broad utility, it is essential to ensure sufficient resources to enable its effective operation. In this context, for AI technologies to provide precise and reliable results, they must have access to extensive, high-quality datasets (Bellini et al., 2022[4]). Furthermore, the quality of the results generated by AI algorithms directly depends on the quality of the input data; thus, inconsistencies or flaws in input data can compromise the support these technologies provide for clinical decision-making (Bellini et al., 2022[4]). Additionally, it is crucial to address data privacy and security concerns to safeguard the integrity and confidentiality of sensitive healthcare information in compliance with the Health Insurance Portability and Accountability Act (HIPAA).

In addition, shared ethics in the use of artificial intelligence (AI) in medicine is an important concern that addresses the responsibility and accountability that arise when technology is involved in medical decision-making, especially in cases of errors (Abdullah et al., 2021[1]). Shared responsibility implies that all parties involved in the creation, implementation, and use of AI have a role in the consequences of errors. Manufacturers and developers of AI, for example, may be held accountable for flaws in the design or accuracy of the system, while physicians must critically evaluate AI recommendations and integrate them judiciously into their clinical decision-making processes (Abdullah et al., 2021[1]).

Despite these challenges, there are numerous opportunities for utilizing generative AI in the pharmaceutical sector, such as analyzing large datasets and identifying patterns that may have previously gone unnoticed (Moulaei et al., 2024[9]). This process can lead to the discovery of new pharmacological targets and the development of more effective drugs, or it can enable personalized medicine by precisely tailoring treatments to individual patients, thereby reducing contraindications, improving outcomes, and minimizing adverse effects (Moulaei et al., 2024[9]).

Overall, generative AI has the potential to significantly impact the pharmaceutical sector through various applications, such as improving drug research and development, transforming personalized treatment, and accelerating clinical trials. However, it is essential to overcome challenges and address ethical concerns to ensure the proper and successful use of generative AI. By adopting this advanced technology, the pharmaceutical industry can foster innovation, improve patient outcomes, and shape the future of healthcare.

## Notes

Lysandro Pinto Borges and Eloia Emanuelly Dias Silva (Department of Biology, Federal University of Sergipe (UFS), Aracaju, Sergipe, Brazil; E-mail: eloiaemanuelly@gmail.com) contributed equally as corresponding author.

## Conflict of interest

None.

## References

[1] Abdullah YI, Schuman JS, Shabsigh R, Caplan A, Al-Aswad LA (2021). Ethics of Artificial Intelligence in medicine and ophthalmology. Asia Pac J Ophthalmol (Phila).

[2] Abrahams E, Downing GJ, Bydon M (2023). On the modern evolution of personalized medicine. The new era of precision medicine. What it means for patients and the future of healthcare.

[3] Ali AM, Mohammed MA (2024). A comprehensive review of artificial intelligence approaches in omics data processing: evaluating progress and challenges. Int J Math Stat Comput Sci.

[4] Bellini V, Valente M, Gaddi AV, Pelosi P, Bignami E (2022). Artificial intelligence and telemedicine in anesthesia: potential and problems. Minerva Anestesiol.

[5] Gangwal A, Ansari A, Ahmad I, Azad AK, Kumarasamy V, Subramaniyan V (2024). Generative artificial intelligence in drug discovery: basic framework, recent advances, challenges, and opportunities. Front Pharmacol.

[6] Gou F, Liu J, Xiao C, Wu J (2024). Research on artificial-intelligence-assisted medicine: A survey on medical artificial intelligence. Diagnostics (Basel).

[7] Johnson KB, Wei W-Q, Weeraratne D, Frisse ME, Misulis K, Rhee K (2021). Precision medicine, AI, and the future of personalized health care. Clin Transl Sci.

[8] Luz A, Ray D (2024). AI-powered disease diagnosis: evaluating the effectiveness of machine learning algorithms. https://easychair.org/publications/preprint/rmqt/open.

[9] Moulaei K, Yadegari A, Baharestani M, Farzanbakhsh S, Sabet B, Afrash MR (2024). Generative artificial intelligence in healthcare: A scoping review on benefits, challenges and applications. Int J Med Inform.

[10] Sharma V, Singh A, Chauhan S, Sharma PK, Chaudhary S, Sharma A (2024). Role of artificial intelligence in drug discovery and target identification in cancer. Curr Drug Deliv.

[11] Thapliyal K, Thapliyal M, Gaur L, Abraham A, Ajith R (2024). AI enhancing digital communication in neurodegenerative disease treatment. AI and neuro-degenerative diseases.

[12] Uddin M, Wang Y, Woodbury-Smith M (2019). Artificial intelligence for precision medicine in neurodevelopmental disorders. NPJ Digit Med.

[13] Vuong QP (2024). The potential for artificial intelligence and machine learning in healthcare: the future of healthcare through smart technologies. Fut Healthcare J.

[14] Wu Y, Xie L (2024). AI-driven multi-omics integration for multi-scale predictive modeling of causal genotype-environment-phenotype relationships. https://arxiv.org/abs/2407.06405.

[15] Zhan C, Tang T, Wu E, Zhang Y, He M, Wu R (2023). From multi-omics approaches to personalized medicine in myocardial infarction. Front Cardiovasc Med.

[16] Zucca S, Nicora G, De Paoli F, Carta MG, Bellazzi R, Magni P (2024). An AI-based approach driven by genotypes and phenotypes to uplift the diagnostic yield of genetic diseases. Hum Genet.

